# Building genomic data structures from compressed representations using prefix-free parsing

**DOI:** 10.1101/gr.281245.125

**Published:** 2026-06

**Authors:** Rahul Varki, Christina Boucher

**Affiliations:** Department of Computer and Information Science and Engineering, Herbert Wertheim College of Engineering, University of Florida, Gainesville, Florida 32611, USA

## Abstract

Advances in high-throughput sequencing have lowered the cost and complexity of genome sequencing, making it possible for the first time to assemble large pangenomic data sets for many species. These data sets, comprising thousands of individuals, already span from hundreds of gigabytes to petabytes, far exceeding the memory capacity of most machines, and are expected to continue growing in scale over time. Already, many traditional bioinformatics tools fail on inputs at this scale because they cannot construct their necessary data structures within memory limits. There is a growing need for methods that can construct these structures directly from compressed representations. Prefix-free parsing (PFP) addresses this challenge. PFP serves as a preprocessing step that compresses sufficiently repetitive text, yet still permits building important data structures for the original data set from its compressed output. This survey offers an overview of PFP, covering its core principles, the primary data structures it enables, current applications, and future research directions.

Before the advent of high-throughput sequencing, obtaining the first complete genome of a species was a costly and time-consuming endeavor. However, advances in sequencing technology have reduced the cost and resources required, enabling the sequencing of thousands of individuals from the same species. This technological leap has led to the generation of large pangenomic data sets across various species. For instance, the 1000 Genomes Project produced over 6 terabytes of assembled genomes (Clarke et al. 2012; The 1000 Genomes Project Consortium 2015), whereas the 100,000 Genomes Project (Turnbull et al. 2018), which focused on sequencing individuals with rare diseases, generated a staggering 21 petabytes worth of data (Weka 2020). The scope of these efforts has extended beyond humans to other species, as seen in the 1001 Genomes Project, which sequenced over a thousand cultivars of the plant *Arabidopsis thaliana* (Weigel and Mott 2009). As a result, the concept of a reference genome has evolved, shifting from a single representative genome per species to a collection of genomes—a pangenome—that provides a more comprehensive and accurate view of the species’ genetic diversity. This shift underscores the need for advanced techniques to efficiently store and process these vast data sets, which has become crucial as pangenomes continue to expand in size and complexity.

Meeting these demands has proven difficult with existing technologies, as most traditional bioinformatics tools cannot scale to handle the volume of pangenomic data. These tools were originally developed for static single-reference genomes, which were the standard at the time. As a result, the underlying methodologies of these tools were not meant to scale to the size of the rapidly growing pangenomic data sets we see today. The lack of scalable, efficient algorithms and data structures remains a critical bottleneck in genomic research.

Among emerging methods, prefix-free parsing (PFP) stands out as a promising algorithm for efficient processing of increasingly large pangenomic data sets. Introduced in Boucher et al. (2019), PFP at a high-level is a compression algorithm that works by segmenting a genomic sequence into overlapping phrases, which can then be used to build data structures in a space-efficient manner. It achieves compression in practice by identifying identical phrases both within and between sequences, exploiting the repetitive nature of pangenomic data sets. Algorithms for building useful data structures from PFP’s output have been developed and continue to evolve. Of particular interest are the PFP-based construction algorithms for the data structures that comprise the FM-index, namely the Burrows–Wheeler transform (BWT) (Burrows and Wheeler 1994) and the suffix array (SA) (Manber and Myers 1993). The FM-index underlies the widely used traditional read aligners BWA (Li 2013) and Bowtie (Langmead et al. 2009; Langmead and Salzberg 2012), but the BWT and SA are also of independent interest. Additionally, the longest common prefix (LCP) array (Manber and Myers 1993) is of interest as it is often built alongside these other data structures as an auxiliary structure. These data structures can be built in space proportional to the output of PFP which in practice is typically smaller than the size of the input when the data are sufficiently repetitive. Although PFP is a relatively recent development, it has already been applied extensively across many applications (see the PFP applications section). PFP has allowed these applications to process some of the largest pangenomic data sets currently available, such as those from the 1000 Genomes Project and similar efforts.

In this survey, we provide a comprehensive review of PFP, focusing on its conceptual foundation, key construction algorithms for important data structures, applications, and future directions. We begin by explaining the core principles of PFP, particularly its use in compressing and indexing repetitive genomic data. We then discuss the BWT, SA, and LCP, along with their corresponding PFP-based construction algorithms. This is followed by a brief overview of other PFP applications, and concludes with potential directions for future PFP-based research. As the size and complexity of genomic data sets continue to grow, methods like PFP offer promising approaches for managing these challenges. Although there is still room for optimization and broader application, PFP provides a solid framework for efficient data processing in genomics. Future innovations in this area will likely further enhance our ability to analyze and interpret pangenomic data, contributing to advances in understanding genetic diversity and disease.

## Overview of prefix-free parsing

In this section, we provide a detailed overview of prefix-free parsing (PFP), which was originally motivated by rsync (Tridgell and Mackerras 1996). Before delving into the specifics, we first present the necessary preliminaries.

### Basic definitions

In this paper, we use 0-based indexing for all strings and arrays. The := symbol denotes an assignment or update operation, the = symbol represents mathematical equality or definition, and the · · · symbol indicates a range of values. Let *T* = *T*[0 · · · *n* − 1] = *T*[0] · · · *T*[*n* − 1] be a finite string over an alphabet Σ of size *σ* whose symbols can be unambiguously ordered. The symbol *T*[*i*] denotes the character at index *i* of *T*, and *T*[*i* · · · *j*] denotes the substring *T*[*i*] · · · *T*[*j*] if 0 ≤ *i* ≤ *j* < *n* or the empty string ϵ otherwise. For 0 ≤ *i* < *n*, the substring *T*[0 · · · *i*] is the (*i* + 1)th prefix of *T*, and *T*[*i* · · · *n* − 1] is the (*n* − *i*)th suffix of *T*. A prefix *T*[0 · · · *i*] is a *proper prefix* if 0 ≤ *i* < *n* − 1, whereas a suffix *T*[*i* · · · *n* − 1] is a *proper suffix* if 0 < *i* ≤ *n* − 1. If *T*_2_[0 · · · *m* − 1] is lexicographically smaller than *T*_1_[0 · · · *n* − 1], then either *T*_2_ is a proper prefix of *T*_1_, or there exists an index 0≤i<min(n,m) such that *T*_2_[0 · · · *i* − 1] = *T*_1_[0 · · · *i* − 1] and *T*_2_[*i*] < *T*_1_[*i*]. Let ${\mathrm{rank}}_{c}(T,i)$ be a function that returns the number of times *c* appears in *T*[0 · · · *i* − 1], where *c* ∈ Σ. Let ${\mathrm{RMQ}}_{A}(i,j)$ denote a range minimum query (RMQ) that returns the minimum value in the subarray *A*[*i* · · · *j*], for 0 ≤ *i* ≤ *j* < |*A*|, where |*A*| is the size of *A*.

The concept of prefix-free is crucial for understanding how the output of prefix-free parsing can be used to build other data structures, so a formal definition is provided.

**Definition 1:**

*A multiset of strings* S *is* prefix-free *if no string in* S *is a proper prefix of another string in* S.

For example, the multiset S={ACCA,GCC,ACCA,TCCAG} is a prefix-free multiset because no string s∈S is a proper prefix of any other string $s^{\prime }\in S$, as the first character of all *unique* strings is lexicographically different.

### Prefix-free parsing

Prefix-free parsing (PFP) is a lossless preprocessing compression algorithm designed for large, repetitive texts such as genomic data sets (Boucher et al. 2019). It achieves effective compression by decomposing the text into variable-length overlapping phrases that often occur. The main advantage of the algorithm is that it enables the scalable construction of other data structures from its compressed output. To understand how the algorithm works in practice, we now turn to its details.

PFP(*T*, *w*, *p*) takes as input a text *T* of length *n* and two user-defined integers greater than 1, denoted *w* and *p*. It first transforms *T* into *T*′ where $T^{\prime }:=\$T{\#}^{w}$ by prepending a $ and appending *w* consecutive copies of #. Both symbols are arbitrary, provided that they are lexicographically smaller than every symbol in Σ. A rolling hash is then computed over all consecutive *w*-length substrings of *T*′ in a single pass by sliding a window of length *w* and hashing each window. Any hash function may be used, provided collisions are rare; in practice, a Karp-Rabin hash (Karp and Rabin 1987) is preferred, as it allows for efficient updates between adjacent windows. This hash is denoted as $H_{p}(t_{w})=H(t_{w})\mathrm{mod}p$, where *H* is the hash function, *p* is the modulus, and *t*_*w*_ is the current substring within the window. Substrings whose hash value is zero are referred to as trigger strings. The following definition succinctly summarizes the set of *w*-length substrings that yield trigger strings.

**Definition 2:**

*Let E be the set of all w-length substrings t_w_ of T such that H_p_(t_w_)* ≡ 0. *We refer to this set of strings as *trigger strings*. In addition, treat* $t_{w}={\#}^{w}$ *as a trigger string, and also consider* $ *as a trigger string even though it is not of length w. We use this set to parse T′ into overlapping phrases*.

Trigger strings identified by the algorithm mark phrase boundaries. As the algorithm scans *T*′, each trigger string signals the end of a phrase (besides the starting $). Each phrase begins at the start of the previous trigger string and ends at the end of the current one; it cannot span any other trigger string. The prepended $ and appended ${\#}^{w}$ characters are always treated as trigger strings, ensuring that the first and last phrase begin and end with a trigger string.

PFP(*T*, *w*, *p*) outputs a dictionary $D_{T}$ and parse $P_{T}$. The dictionary stores only the unique phrases encountered, while the parse records the order that those phrases occur in the text. During parsing, when a trigger string is identified, the algorithm adds the newly terminated phrase to $D_{T}$ if it is not already present, but always appends a reference to the corresponding dictionary entry to $P_{T}$. After parsing, PFP lexicographically sorts the phrases in $D_{T}$ and updates the references in $P_{T}$ with the corresponding dictionary integer ranks before writing the files to disk. The combined output size depends on the repetitiveness of the text and the selection of *w* and *p*. Lucà et al. (2025) extensively tested PFP with various configurations of *w* and *p* on different biological data sets and found that, for most data sets, fixing *w* while increasing *p* leads to decreased output size, reaching a minimum when *p* = 50 or *p* = 100. Beyond that, they observed a slight increase in output size, likely due to longer dictionary phrases caused by fewer observed trigger strings. Reconstructing *T* from $D_{T}$ and $P_{T}$ is straightforward. The $D_{T}$ phrases need to be appended in the order specified by $P_{T}$, trimming the last *w* characters of each phrase to account for the overlap.

**Figure 1.**
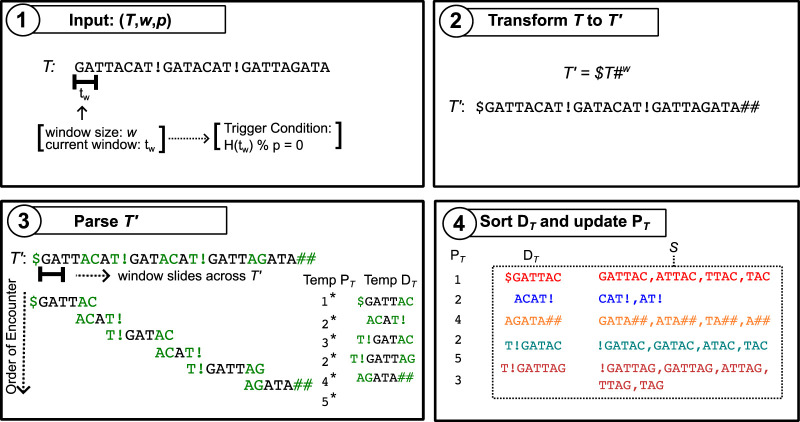
Overview of the PFP workflow. (1) PFP takes as input a text *T* and two integer parameters, the window size *w* and the hash modulus *p*. In the example, *w* = 2 and *p* ∈ *Z*^+^, where *Z*^+^ represents the set of positive integers. (2) It internally prepends the $ symbol and appends *w* consecutive copies of # to *T*. (3) The modified text is parsed by sliding a window of length *w* across the text; when a trigger string is encountered (highlighted in green), the current phrase terminates and the next phrase begins. During parsing, the dictionary $D_{T}$ and parse $P_{T}$ are constructed, with $D_{T}$ initially unsorted and $P_{T}$ containing temporary phrase records (*). (4) Finally, $D_{T}$ is sorted and the records in $P_{T}$ are replaced by the ranks of the corresponding dictionary phrases; the dotted box shows the multiset *S* of suffixes longer than *w* derived from phrases in $D_{T}$.

The name of the algorithm, prefix-free parsing, stems from the observation that all dictionary phrase suffixes longer than *w* are prefix-free with respect to one another (see Definition 1). This is formalized in Lemma 3, which we now prove. Let S be the multiset of all dictionary phrase suffixes that are longer than *w*. Each s∈S must span the end trigger string of its respective phrase, and this trigger string cannot be a prefix of *s*, because all suffixes are longer than *w*. If the suffixes in S were not prefix-free with respect to each other, then some s∈S would be a proper prefix of another $s^{\prime }\in S$. This would imply that the phrase from which *s*′ originates spans a trigger string that is neither a proper prefix nor a suffix of the phrase, contradicting the definition of a PFP phrase. Therefore, S must be prefix-free. Note that although S is prefix-free, it does not follow that each s∈S is necessarily unique, as distinct phrases may share the same suffix. An overview of the entire PFP workflow is provided in Figure 1.

**Lemma 3 (Boucher et al. 2019):**

*Given a text T and* $PFP(T,w,p)=D_{T},P_{T},$ *we denote the multiset of all phrase suffixes in* $D_{T}$ *that have a length greater than w as* S. *It follows that* S *is a prefix-free multiset, which means that no string in* S *is a proper prefix of any other string in* S.

We illustrate PFP using a small example. Instead of the one-pass algorithm presented earlier, we present it as a two-pass approach in this example for the sake of clarity. Let *w* = 2 and

$$T^{\prime }:=\$T\#\#=\$GATTACAT!GATACAT!GATTAGATA\#\#.$$

Next, imagine we do one pass through *T*′ and we use a Karp-Rabin hash that defines the set E of trigger strings in *T*′ as E={AC,AG,T!,$,##}. In the second pass, knowing these trigger strings, we would see that the dictionary D is

{$GATTAC,ACAT!,AGATA##,T!GATAC,T!GATTAG}

and the parse P is [1, 2, 4, 2, 5, 3].

The multiset of suffixes S as specified in Lemma 3 would be

{$GATTAC,GATTAC,ATTAC,TTAC,TAC,ACAT!,CAT!,AT!,AGATA##,GATA##,ATA##,TA##,A##,

T!GATAC,!GATAC,GATAC,ATAC,TAC,T!GATTAG,!GATTAG,GATTAG,ATTAG,TTAG,TAG}.

PFP is designed for large repetitive texts, so this small example does not fully demonstrate the algorithm’s compression capabilities. In practice, the combined representation of $D_{T}+P_{T}$ is usually much smaller than *T* when *T* is large and repetitive. However, even in this small example, PFP achieves a level of compression. The second dictionary index appears twice in the parse, indicating that the algorithm effectively eliminated storing one instance of the second dictionary phrase without penalty. In practice, PFP is fairly efficient. The algorithm uses $O(|D_{T}|+|P_{T}|)$ memory and runs in O(n) time, where $\left|D_{T}\right|$ represents the length of all the phrases in $D_{T}$, $\left|P_{T}\right|$ is the length of $P_{T}$, and *n* is the length of *T*. PFP can be used as a preprocessing step to build data structures such as ${BWT}_{T}$, ${SA}_{T}$, and ${LCP}_{T}$. In the following sections, we will go over the construction algorithms of these data structures.

### Recursive prefix-free parsing

Recursive PFP provides an additional layer of compression on the PFP output. For large repetitive data sets, the dictionary $D_{T}$ has been observed to grow sublinearly relative to the parse $P_{T}$ (Oliva et al. 2023). Recall from the Prefix-free parsing section that $D_{T}$ grows only when a unique phrase is encountered, whereas $P_{T}$ grows with every phrase. Although the output of PFP is usually much smaller than the input, it can still be too large for memory-constrained applications, prompting the development of recursive PFP.

Recursive PFP applies PFP to the parse $P_{T}$, producing a new dictionary $D_{P}$ and parse $P_{P}$ that fully represent it. Combined with $D_{T}$, these components fully represent *T* and form the output of recursive PFP. The key idea behind recursive PFP is that although $P_{T}$ grows linearly, it remains highly repetitive, as phrases typically occur in the same order in repetitive texts, making it suitable for another round of PFP. Once PFP is applied to $P_{T}$, it can be safely deleted. If $P_{T}$ is larger than $D_{T}$ and is compressible, then recursive PFP will produce a noticeably smaller output than PFP. The idea of recursive PFP was briefly mentioned in Gagie et al. (2019) and more recently in Oliva et al. (2023). In theory, any application using PFP outputs can also be modified to use recursive PFP outputs. See Table 1 for applications where the recursive PFP has been successfully applied. Recursive PFP enables PFP to scale to arbitrarily large repetitive data sets by applying it recursively to each successive parse, provided the parse remains sufficiently compressible.

**Table 1.** Applications of prefix-free parsing

Category	Subcategory	Paper	Contribution
Data structure	BWT, SA, LCP	Boucher et al. (2019), Kuhnle et al. (2020), and Rossi et al. (2022)	Builds the BWT, SA, and LCP using PFP
	BWT, SA, LCP	Oliva et al. (2023) and Ferro et al. (2024)	Builds the BWT, SA, and LCP using recursive PFP
	eBWT	Boucher et al. (2021a, 2024)	Computes the extended BWT (eBWT) for a collection of strings using PFP
	BWT	Díaz-Domínguez et al. (2025)	Shows how to merge BWTs built with PFP
	Compressed suffix tree	Boucher et al. (2021b) and Oliva et al. (2022)	Shows how PFP output can be modified to support full suffix tree functionality
String matching	MEMs	Rossi et al. (2022) and Boucher et al. (2024)	Computes maximal exact matches (MEMs) using data structures built with PFP
	MEMs, MUMs	Shivakumar and Langmead (2025)	Computes maximal exact (MEM) or unique (MUM) matches across sequences using data structures built with PFP
	MEMs, full read alignment	Varki et al. (2025)	MEM-based short-read aligner built on index constructed with PFP
	MS	Boucher et al. (2021c) and Rossi et al. (2022)	Computes matching statistics (MS) with data structures built with PFP
	PMLs	Ahmed et al. (2021, 2023)	Computes an approximation of MS called pseudomatching lengths (PMLs) using data structures built from PFP
	LF-mapping, BWT	Hong et al. (2024)	Shows that leveraging PFP phrases for LF-mapping yields better performance than standard character-level LF-mapping
Compression	LZ77	Hong et al. (2023)	Constructs LZ77 factorization using PFP
	RePair	Gagie et al. (2019)	Builds RePair grammar from PFP output
	RePair	Kim et al. (2024)	Builds RePair grammar from recursive PFP output

The table highlights a *subset* of works that use PFP, categorized by their primary contribution. Although grouped by category, many of these papers build on one another, resulting in significant overlap.

## Building classic bioinformatics data structures from PFP

This section first introduces the BWT, SA, and LCP array data structures, followed by a description of how to construct them using the PFP components.

### Burrows–Wheeler transform (BWT)

**Figure 2.**
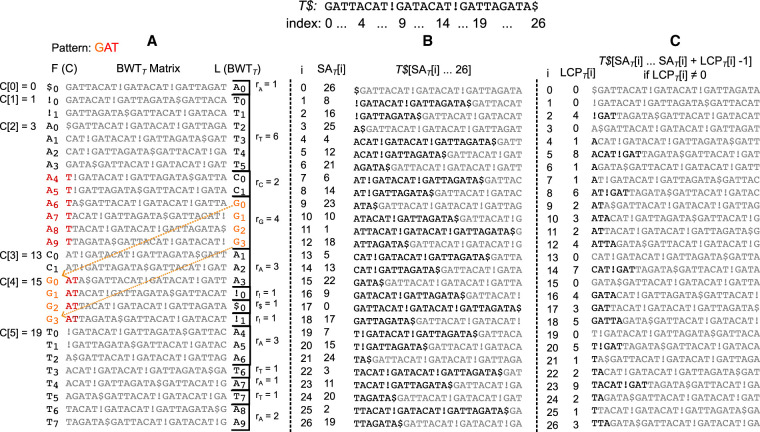
Visualization of the ${BWT}_{T}$, ${SA}_{T}$, and ${LCP}_{T}$ for the example introduced in the Prefix-free parsing section. For clarity, the ${BWT}_{T}$ matrix is shown in each subfigure to highlight the information captured by each data structure. (*A*) The ${BWT}_{T}$. In the ${BWT}_{T}$ matrix, only the first (*C*) and last (${BWT}_{T}$) columns are highlighted; these columns are annotated with character ranks as subscripts, although in practice these ranks are computed using an auxiliary data structure. To the *left* of the first column, we illustrate how the *C* array is populated, and to the *right* of the last column we show the maximal character runs in the ${BWT}_{T}$. Also shown is how pattern matching is performed using the *C* array and ${BWT}_{T}$. (*B*) The ${SA}_{T}$. In the ${BWT}_{T}$ matrix, the suffixes of *T*$ that prefix each rotation are highlighted. (*C*) The ${LCP}_{T}$. In the ${BWT}_{T}$ matrix, the longest common prefix between consecutive rotations of *T*$ is highlighted.

The Burrows–Wheeler transform (BWT) was introduced in Burrows and Wheeler (1994) as a reversible preprocessing technique that rearranges text to make it more compressible. The ${BWT}_{T}$ is a special reversible permutation of the text *T*$, where *T*[0 · · · *n* − 1] is the original text and $ is a unique sentinel character appended to *T* that is lexicographically smaller than all other characters. Conceptually, the ${BWT}_{T}$ can be constructed by first building the ${BWT}_{T}$ matrix by sorting all rotations of *T*$ lexicographically. The last column of the ${BWT}_{T}$ matrix is referred to as the ${BWT}_{T}$. To illustrate this construction, Figure 2A shows a visualization of ${BWT}_{T}$ for the example introduced in the Prefix-free parsing section. However, in practice, the ${BWT}_{T}$ matrix is never explicitly constructed due to its quadratic space requirements.

The key observation of Burrows and Wheeler is that identical characters tend to group together and create *runs* in the ${BWT}_{T}$. For example, the text used in Figure 2A contains 27 characters, yet its ${BWT}_{T}$ has only 13 maximal character runs. This demonstrates how the ${BWT}_{T}$ reorders *T*$ to increase its local regularity by exploiting higher-order contexts. The effect arises because the initial lexicographic sort groups together rotations with similar or identical prefixes. In particular, the start of each rotation corresponds to a suffix of *T*$, which explains why the prefixes of consecutive rotations reveal repeated substrings in the text. Because texts are structured for comprehensibility, only a few distinct characters typically precede any given substring, and this number decreases as the length of the substring increases. This structural property gives rise to the runs observed in the ${BWT}_{T}$. The presence of runs in the ${BWT}_{T}$ does not compress the text on its own, but the resulting structure is well-suited for compression by other algorithms. The authors suggest applying a move-to-front transformation (Bentley et al. 1986) to the ${BWT}_{T}$ to convert these runs into a skewed distribution of small integers, followed by Huffman encoding (Huffman 1952). This compression scheme forms the core of the Bzip2 algorithm (Seward 1996), a widely used compressor today.

Another important property of the ${BWT}_{T}$ is that it is reversible, allowing the original text to be recovered. This reversibility is guaranteed by the sentinel character $ appended to the text, which establishes a unique total order among the rotations of *T* by breaking ties that might otherwise arise during lexicographic comparison. Because all rotations of *T*$ are used to construct the ${BWT}_{T}$, every row and column of the conceptual ${BWT}_{T}$ matrix is a permutation of *T*$, as illustrated in Figure 2A. In particular, the relative order of the characters in *T*$ is preserved between the first and last columns because the rotations are lexicographically sorted. Moving from the last column to the first effectively steps back one character in *T*$. This relationship between the two columns is known as the last-to-first (LF) mapping, formally defined below.

**Definition 4:**

*Let C be an integer array where C*[*c*] *stores the number of characters in T*$ *lexicographically smaller than c. Then, the LF-mapping property is defined as* $LF(i,c):=C[c]+{rank}_{c}({BWT}_{T},i)$ *and* $LF(i):=LF(i,{BWT}_{T}[i])$.

From Definition 4, *LF*(*i*) returns the index in the first column of the ${BWT}_{T}$ matrix that corresponds to the character at index *i* in the ${BWT}_{T}$. The *C* integer array is a highly compressed representation of the first column of the ${BWT}_{T}$ matrix, a column whose characters are lexicographically sorted. Both the first column (*C*) and the last column (${BWT}_{T}$) are essential for LF-mapping. However, compared to the size of the ${BWT}_{T}$ which is the size of |*T*$|, the *C* array is significantly smaller and requires only |Σ| + 1 entries. For DNA, this means only four entries for the different nucleotides, plus one entry for the special $ character appended to the text.

The text *T*$ can be reconstructed from the ${BWT}_{T}$ by repeatedly applying *LF*(*i*), starting from the last character, $. Let *T*′ represent the reconstructed text. Initialize *T*′[*n*] := $ and *s* := 0 where *n* is the length of *T* and *s* is an iterator where 0 ≤ *s* ≤ *n*. For *i* = *n* − 1, …, 0, assign $T^{\prime }[i]:={BWT}_{T}[s]$ and update *s* := *LF*(*s*). At the end of the loop, we will get *T*′ = *T*$, recovering the text. In other words, *T*$ was reconstructed in reverse using the LF-mapping property.

Ferragina and Manzini (2000) showed that the ${BWT}_{T}$ can be used for more than just compression purposes. In particular, they demonstrated that the ${BWT}_{T}$ enables efficient exact backward pattern matching within the text. This process is similar to how the original text is reconstructed. However, instead of tracking individual positions, the algorithm maintains a range in the ${BWT}_{T}$ corresponding to all suffixes of *T* that match the current query suffix. Given a pattern *P*[0 · · · *p* − 1], backward matching consists of *p* steps that preserve the following property: after matching the last *i* characters of *P*, the start of the search range *sp* corresponds to the first row of the ${BWT}_{T}$ matrix prefixed by *P*[*p* − *i* · · · *p* − 1] and the end of the range *ep* corresponds to the last such row. To extend the match to the next preceding character, LF-mapping is applied to both *sp* and *ep*, computing the new range as:

$$\begin{array}{rl} sp: & =C[c]+{\mathrm{rank}}_{c}({BWT}_{T},sp),\\ ep: & =C[c]+{\mathrm{rank}}_{c}({BWT}_{T},ep+1)-1, \end{array}$$

where *c* is the character at *P*[*p* − *i* − 1]. Note that if the rank queries of both *sp* and *ep* return the same value during the matching, the pattern does not occur in the text. An example of pattern matching is shown in Figure 2A. Also, observe that we can only determine the number of occurrences of a pattern with just the ${BWT}_{T}$. To find their locations within the text requires augmenting the ${BWT}_{T}$ with another data structure called the suffix array.

### Suffix array

The suffix array (SA) was formally introduced in Manber and Myers (1993) as a space-efficient alternative to suffix trees (Weiner 1973; McCreight 1976; Apostolico 1985) for online string searches, demonstrating three to five times lower space usage. Conceptually simple, the ${SA}_{T}$ is an array of length |*T*$| where ${SA}_{T}[i]$ gives the starting index in the text of the (*i* + 1)th lexicographically smallest suffix of *T*$, for 0 ≤ *i* < |*T*$|. That is, the ${SA}_{T}$ stores the starting indices of the lexicographically sorted suffixes of *T*$. Figure 2B shows the ${SA}_{T}$ for the example introduced in the Prefix-free parsing section. Although the ${SA}_{T}$ is more space-efficient than the suffix tree, it still requires O(|*T*$|) space.

The ${SA}_{T}$ and ${BWT}_{T}$ are intrinsically linked, as both are constructed by lexicographically sorting the suffixes of the text *T*$. More formally, the relationship between the two data structures is as follows:

$${BWT}_{T}[i]:=T\$[({SA}_{T}[i]-1)\mathrm{mod}(n+1)],$$

where *T*$[*n*] = $. Most implementations of the ${BWT}_{T}$ require building the ${SA}_{T}$ first. This relationship between the two data structures was already known when Burrows and Wheeler published their seminal paper on the BWT (Burrows and Wheeler 1994). Later, Ferragina and Manzini showed that augmenting the ${BWT}_{T}$ with the ${SA}_{T}$ allows efficient retrieval of pattern occurrence indices (Ferragina and Manzini 2000). However, rather than storing the full ${SA}_{T}$, they proposed a sampled version that stores only a subset of suffix indices. In practice, this saves memory at the cost of slower locate queries. If the index of the matched suffix is stored in the sampled ${SA}_{T}$, it can be returned immediately. Otherwise, LF-mapping is applied iteratively until a stored value is reached, from which the index of the matched suffix can be recovered. Let *s* be the index of the suffix prefixed by the pattern whose value is not stored, and let *s*′ be the index of the next suffix with a stored value, found after the *v* LF-mapping steps. Then,

$${SA}_{T}[s]:=({SA}_{T}[s^{\prime }]+v)\mathrm{mod}(n+1).$$

The combination of the ${BWT}_{T}$ and a sampled ${SA}_{T}$ is known as the FM-index (Ferragina and Manzini 2000, 2005). This index is used by popular read aligners such as Bowtie (Langmead et al. 2009; Langmead and Salzberg 2012) and BWA (Li 2013).

### The r-index

The FM-index, which consists of the ${BWT}_{T}$ and ${SA}_{T}$, has been a foundational data structure in bioinformatics. However, a notable drawback is that the size of both the ${BWT}_{T}$ and ${SA}_{T}$ is proportional to the size of *T*$ (see the BWT and SA sections). For very large data sets, these data structures become too large to be practical. As a result, considerable research has focused on compressing the FM-index. The use of run-length encoding the ${BWT}_{T}$ has been established for some time (Mäkinen and Navarro 2005; Mäkinen et al. 2010), but sampling the ${SA}_{T}$ within similar space remained an open problem for much longer. The problem remained open until 2017, when Gagie et al. (2020) introduced the *r*-index in their preprint, solving the problem by storing ${SA}_{T}$ values only at the run boundaries of the ${BWT}_{T}$. With a few small auxiliary data structures, the missing ${SA}_{T}$ can be efficiently computed. This insight completed the design of the *r*-index, enabling it to be stored in O(r) space, where *r* denotes the number of maximal character runs in the ${BWT}_{T}$. In practice, *r* ≪ |*T*$|. Despite the shift toward the *r*-index in recent literature, its construction is conceptually rooted in the FM-index, which serves as the architectural basis for its compressed successor.

### Building the FM-index (BWT and SA) from PFP

**Figure 3.**
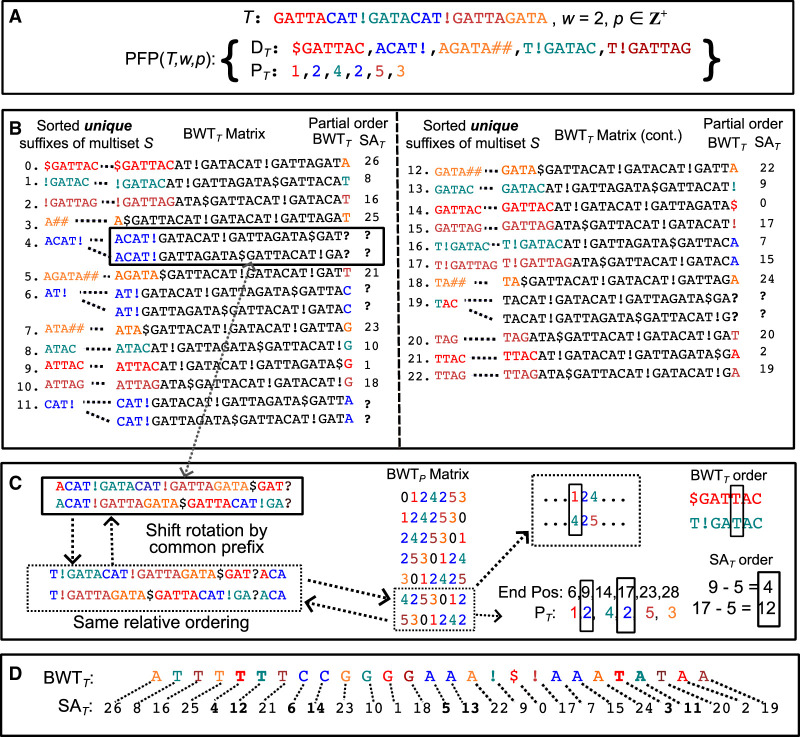
PFP-based construction of the FM-index for the example introduced in the Prefix-free parsing section. Color denotes phrase membership in all subfigures. (*A*) The input text and the PFP output after parsing. See the Prefix-free parsing section for more details. (*B*) The partial order ${BWT}_{T}$ and ${SA}_{T}$ constructed from $D_{T}$ and the occurrence frequencies of $P_{T}$, where each unique suffix in *S* (derived from $D_{T}$) prefixes at least one ${BWT}_{T}$ rotation and appears in sorted order in the matrix. Suffixes that prefix multiple rotations create ambiguous entries, denoted by a bold “?” symbol. (*C*) Ambiguous entries are resolved using the positions, in the ${BWT}_{P}$, of the phrases from which those suffixes originate. (*D*) The total order ${BWT}_{T}$ and ${SA}_{T}$ computed after resolving all ambiguous entries. Entries that were previously ambiguous are bolded.

Boucher et al. (2019) introduced the PFP algorithm as a preprocessing step for the scalable construction of large BWTs. Building on this work, Kuhnle et al. (2020) showed how to construct the ${SA}_{T}$ sample alongside the ${BWT}_{T}$. In doing so, they effectively showed how to construct the FM-index of the text from the PFP output, namely $D_{T}$ and $P_{T}$. We illustrate this process through a detailed walkthrough of the algorithm by continuing the example introduced at the end of the Prefix-free parsing section, which serves as the basis for the following discussion. Figure 3A shows the PFP output.

In the Prefix-free parsing section, we described how PFP is applied to the text $\$T{\#}^{w}$. The prepended $ and appended ${\#}^{w}$ ensure that the text begins and ends with a trigger string. Although $ is shorter than the standard *w*-length trigger string, it still functions as a valid trigger because it is guaranteed to be unique within the text. This uniqueness preserves the prefix-free property (see Lemma 3). Furthermore, this use of $ is consistent with its role in the standard BWT and SA constructions, where $ is the lexicographically smallest sentinel appended to *T*. Establishing this consistent role of $ is crucial, as it allows us to align the lexicographically sorted rotations of the PFP modified text $\$T{\#}^{w}$ with those of the standard FM-index text *T*$.

Structurally, *T*$ and $\$T{\#}^{w}$ differ in only two respects: the placement of the sentinel symbol $ and the addition of the appended ${\#}^{w}$ characters. The placement of the sentinel, whether at the beginning or end, does not affect the lexicographic ordering of rotations, as the text is treated circularly; this is consistent with the definition of the BWT and SA, which are constructed from all cyclic rotations of the text. Similarly, appending ${\#}^{w}$ symbols does not alter the ordering because these symbols are defined to be lexicographically smallest, ensuring the lexicographical order of the rotations is preserved. Taken together, these observations imply that the lexicographical ordering of the rotations for both texts is identical. Furthermore, all suffixes of *T*$ act as prefixes to rotations of $\$T{\#}^{w}$ when the sentinel is conceptually moved to the front. Therefore, the ability to lexicographically sort $\$T{\#}^{w}$ implies that *T*$ can also be sorted, effectively bridging the two texts.

The lexicographically sorted rotations of $\$T{\#}^{w}$ can be obtained by sorting the dictionary phrase suffixes of the multiset *S*. From Lemma 3, we know that the suffixes in this multiset have length greater than *w* and are prefix-free, meaning that the *unique* suffixes in the multiset can be unambiguously ordered lexicographically. Because the initial PFP segmentation is lossless and divides the text into *w*-length overlapping phrases, this means that each suffix in *S* must be a prefix to at least one rotation of the text $\$T{\#}^{w}$, as illustrated in Figure 3B. By transitivity, lexicographically ordering the unique suffixes in *S* induces the corresponding lexicographic ordering of *T*$. However, Figure 3B shows only 23 unique suffixes, whereas Figure 2B shows that *T*$ contains 27 suffixes, revealing a discrepancy of four suffixes. There are two reasons for the discrepancy in the counts. First, the second dictionary phrase appears twice in the parse, so each of its suffixes longer than *w* is a prefix to twice as many rotations as it would if the phrase appeared only once. This accounts for three of four missing suffixes. Second, as shown in Figure 3B, the 20th lexicographically smallest suffix (index 19), TAC, is shared by the first and fourth dictionary phrases. Although TAC prefixes two distinct rotations in the text, these occurrences are represented by a single entry in the sorted list of unique suffixes, accounting for the final missing suffix. This information can be derived solely from the dictionary and the frequency of each phrase in the parse. However, relying solely on this information yields only a partial ordering of the rotations, resulting in an incomplete ${BWT}_{T}$ and ${SA}_{T}$, as shown in Figure 3B. If a phrase appears multiple times, we cannot determine which phrase precedes each occurrence, making it difficult to fully resolve the ${BWT}_{T}$ and ${SA}_{T}$. Similarly, if multiple phrases share a common suffix, the ${BWT}_{T}$ character and ${SA}_{T}$ value for the rotations prefixed by that suffix become ambiguous. Computing the total order ${BWT}_{T}$ and ${SA}_{T}$ requires positional information from the parse.

Ambiguities in the partial order ${BWT}_{T}$ and ${SA}_{T}$ can be resolved by examining the ${BWT}_{P}$, as shown in Figure 3C. Recall that the parse lists the lexicographic ranks of dictionary phrases in textual order. Consequently, the sorted rotations of the parse correspond to the relative order of the phrases within the sorted rotations of the text. Because ambiguities in the partial order arise from identical phrase suffixes, they can be resolved by comparing the text immediately following those suffixes (the remainders of the rotations). Because PFP phrases always end at trigger strings, the remainders of the rotations correspond to the start of the *next* phrase in the sequence. Therefore, the lexicographical ordering of the parse rotations (captured by the ${BWT}_{P}$) provides the necessary information to resolve these ambiguities.

Continuing from the partial order in our running example, the first ambiguity arises because the second phrase, ACAT#, is preceded by both the first and fourth dictionary phrases. Therefore, to determine the ordering, we must identify which occurrence of the second phrase appears earliest in the ${BWT}_{P}$. In the parse sequence, these occurrences are followed by the fourth and fifth phrases, respectively. A visual inspection of the first column of the ${BWT}_{P}$ matrix confirms that the fourth phrase appears earlier than the fifth. This implies that the second phrase occurrence preceded by the first phrase is lexicographically smaller than the one preceded by the fourth phrase. In the context of resolving the ${BWT}_{T}$, it coincidentally does not matter because both preceding phrases end with T (after accounting for the *w*-length overlap); however, it does matter in resolving the ${SA}_{T}$ at these indices. The second ambiguity arises because the suffix TAC is shared by both the first and fourth dictionary phrases. Because it is preceded by different characters in each phrase, the order in which the phrases appear in the ${BWT}_{P}$ becomes significant in constructing the ${BWT}_{T}$. To resolve the ambiguity, we follow the same steps as before. It turns out that both phrases are followed by occurrences of the second phrase, whose order we previously determined. By transitivity, this implies that the first phrase precedes the fourth, meaning that T should precede A in the ${BWT}_{T}$ for these specific rotations. Finally, once the correct ordering is determined, calculating the ${SA}_{T}$ values is straightforward: the suffix length is subtracted from the corresponding phrase’s end position in the text to obtain the suffix’s start position. The total order ${BWT}_{T}$ and ${SA}_{T}$ can be seen in Figure 3D.

To efficiently implement these concepts, two technical optimizations are required. First, the ordering of the suffixes in *S* in practice is achieved by constructing the suffix array of the dictionary, denoted as ${SA}_{D}$. The dictionary phrases are concatenated using a unique end-of-word separator symbol [EOW], forming the string $D_{T}=d_{1}[\mathrm{EOW}]d_{2}[\mathrm{EOW}]\cdots d_{|D|-1}[\mathrm{EOW}]d_{\left|D\right|}[\mathrm{EOW}]$ where *d*_*i*_ is the *i*th lexicographically sorted dictionary phrase. In ${SA}_{D}$, any suffix containing [EOW] within its first *w* + 1 characters is ignored because these suffixes are shorter than or equal to *w* and therefore do not belong to multiset *S*. The [EOW] symbol is defined to be lexicographically smaller than all other characters, including the $ and # symbols added during the PFP parsing. Therefore, the first —$D_{T}$—suffixes in ${SA}_{D}$ will correspond to rotations that start with [EOW]. Notably, the [EOW] symbols will appear in the exact positional order of their occurrence in the concatenated $D_{T}$, because the dictionary phrases were previously sorted. This structure is beneficial, as it allows for binary search in this subsection of the ${SA}_{D}$ to efficiently identify the originating phrase of any suffix and calculate its length. Second, explicitly scanning the ${BWT}_{P}$ is inefficient. Instead of explicitly scanning the ${BWT}_{P}$, an auxiliary data structure called the inverted list (IL) is built. This is implemented as a single array of size $|P_{T}|+1$ (accounting for the parse length $\left|P_{T}\right|$ plus the sentinel). The array stores the ${BWT}_{P}$ indices of all phrase occurrences, grouped by phrase and ordered lexicographically. In this capacity, the IL array effectively acts as an interface between the first column (sorted phrases) and the last column (${BWT}_{P}$) of the ${BWT}_{P}$ matrix, enabling the efficient retrieval of phrase occurrences in the ${BWT}_{P}$ without the overhead of a full scan. These indices can then be used to index auxiliary data structures of size $|P_{T}|+1$ generated by PFP, which store the ${BWT}_{T}$ character and ending position of each phrase to efficiently resolve ambiguous entries in the ${BWT}_{T}$ and ${SA}_{T}$.

### Longest common prefix array

The longest common prefix (LCP) array was introduced in Manber and Myers (1993) as an auxiliary structure to accelerate pattern search with the SA. For two arbitrary strings *T*_1_[0 · · · *n* − 1] and *T*_2_[0 · · · *m* − 1], the value of $LCP(T_{1},T_{2})$ falls into one of the three cases: (1) 0, if *T*_1_[0] ≠ *T*_2_[0], (2) min(n,m), if *T*_1_ or *T*_2_ prefixes the other, or (3) the unique *i* such that *T*_1_[0 · · · *i* − 1] = *T*_2_[0 · · · *i* − 1] and *T*_1_[*i*] ≠ *T*_2_[*i*]. The ${LCP}_{T}$ array stores the LCP values between consecutive suffixes of *T* in lexicographic order. Formally, ${LCP}_{T}[0\cdots n]$ is defined by ${LCP}_{T}[0]:=0$ and for 1 ≤ *i* ≤ *n*:

$${LCP}_{T}[i]:=\mathrm{LCP}(T\$[{SA}_{T}[i-1]\cdots n],T\$[{SA}_{T}[i]\cdots n]),$$

where *T*$ [*n*] = $. The ${LCP}_{T}$ array for the example introduced in the Prefix-free parsing section is shown in Figure 2C. One useful property of the ${LCP}_{T}$ array is that the longest common prefix of *any* two suffixes can be computed by performing a range minimum query (RMQ) over the ${LCP}_{T}$ values between their indices in the suffix array. More formally, for two indices *i* < *j*, computing LCP(i,j) is equivalent to finding the minimum value in the range ${LCP}_{T}[i+1\cdots j]$. This query is often referred to as computing the longest common extension (LCE) between two suffixes of *T*. For example, in Figure 2C, the LCP value between ${SA}_{T}[7]$ and ${SA}_{T}[12]$ can be found by computing ${RMQ}_{{LCP}_{T}}(8,12)=$ $\min({LCP}_{T}[8],{LCP}_{T}[9],{LCP}_{T}[10],{LCP}_{T}[11],{LCP}_{T}[12])=2$, which corresponds to the common prefix AT. There exist data structures that support constant O(1) time RMQ queries that can be built in linear time (Fischer 2010). Precomputing the ${LCP}_{T}$ array helps avoid performing redundant character comparisons, making it especially useful in string matching problems.

### Building the LCP array from PFP

**Figure 4.**
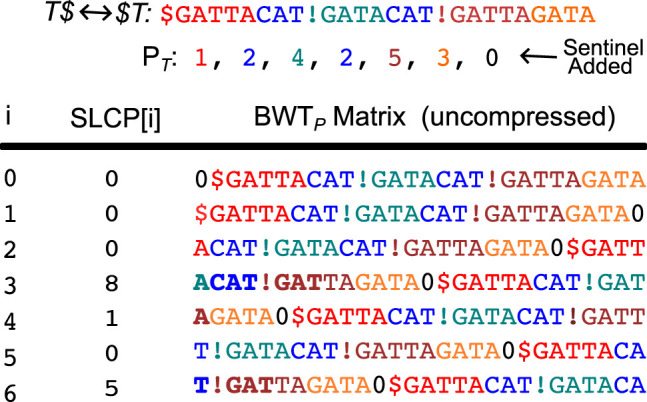
Visualization of the SLCP for the example introduced in the Prefix-free parsing section. The SLCP represents the ${LCP}_{P}$ with $P_{T}$ decompressed and the last *w* characters trimmed from each phrase, effectively creating rotations starting at phrase boundaries of *T*. In the accompanying ${BWT}_{P}$ matrix, colors are used to highlight phrases within each rotation, and bolded characters emphasize the SLCP values. Because the built-in overlap is removed, the last *w* characters of each phrase are split and colored half by the preceding phrase and half by the following phrase.

Rossi et al. (2022) introduced a PFP-based FM-index construction that simultaneously computes the LCP array, allowing for a wider range of string queries that can be performed with the index. To construct the complete ${LCP}_{T}$ array, they first build an auxiliary structure called the SLCP, a variant of the ${LCP}_{P}$, where the LCP values are computed over the uncompressed form of $P_{T}$0, excluding the *w*-length overlap between phrases. Note that the 0 is a sentinel appended to $P_{T}$, which is why the dictionary ranks in the parse start at 1 rather than 0. More formally, $SLCP[0\cdots |P_{T}|]$ is defined by SLCP[0⋯2]=0 and for $2<i\leq |P_{T}|$:

$$SLCP[i]:=LCP(T\$[p_{i-1}\cdots n],T\$[p_{i}\cdots n]),$$

where *T*$ [*n*] = $ and *p*_*i*−1_, *p*_*i*_ are the starting indices in *T*$ of the rotations that are prefixed by the (*i* − 1)th and *i*th lexicographically smallest suffixes of the uncompressed $P_{T}$. The initialization accounts for the two sentinels: the text sentinel $ and the parse sentinel 0, which occupy the initial lexicographical ranks. This data structure can be computed in $O(|P_{T}|)$ time with a slight modification of the algorithm described in Kasai et al. (2001). Figure 4 shows an image of the SLCP computed from the parse of the example text introduced in the Prefix-free parsing section. Additionally, they build a RMQ data structure over the SLCP.

During the FM-index construction, the ${LCP}_{T}$ can start to be built once the lexicographic ordering of the rotations is determined. As discussed in the Building the FM-index from PFP section, the problem of sorting the rotations reduces to correctly sorting the suffixes of the $D_{T}$ phrases with length greater than *w*, which prefix all rotations. Now let *α* and *β* be the $D_{T}$ suffixes that prefix the rotations of ${SA}_{T}[i-1]$ and ${SA}_{T}[i]$ and let *p*_1_, *p*_2_ be the indices in the ${BWT}_{P}$ of the phrases from which *α* and *β* are derived from and let *p*_1_ < *p*_2_. Then, the ${LCP}_{T}$ can be computed as follows:

$${LCP}_{T}[i]:=\left\{ \begin{array}{cc} \text{LCP}(\alpha ,\text{β})\; & \text{if}\;\alpha \neq \text{β},\;\\ |\alpha |-w+h\; & \text{otherwise},\; \end{array}\right.$$

where $h={RMQ}_{SLCP}(p_{1}+1,p_{2})=\min(SLCP[p_{1}+1\cdots p_{2}])$. If the suffixes that prefix two consecutive sorted rotations differ, their LCP value can be computed directly by comparing the suffixes, as the difference must be within their length due to the prefix-free property of the multiset *S*. However, if the suffixes that prefix both rotations are identical, then additional information is needed to compute the LCP value. Recall that $P_{T}$ is effectively a compressed representation of *T*$ and that the rotations of $P_{T}$ represent a subset of the rotations of *T*$. Because $P_{T}$ encodes the lexicographic ranks of phrases in $D_{T}$, its sorted rotations appear in the same relative order as in *T*$. If two rotations share the same $D_{T}$ suffix as a prefix, then the remainder of each rotation must begin with a full dictionary phrase, implying that the remainders of these rotations correspond to a rotation of $P_{T}$. As discussed in the LCP section, we can compute the LCP value between these rotations by performing an RMQ on the SLCP between indices *p*_1_ and *p*_2_. This is equivalent to performing an RMQ on the ${LCP}_{T}$ array between the ${SA}_{T}$ indices of these rotations. This enables the computation of the remaining match length between the rotations.

Suppose that we want to calculate ${LCP}_{T}[23]$ in our example. Recall that in the Prefix-free parsing section that this rotation was initially ambiguous in the partial order ${BWT}_{T}$ because it was prefixed by the suffix TAC, which is shared by both the first and fourth dictionary phrases. We now know that the rotation of the first phrase precedes the rotation from the fourth phrase. Because these two rotations are prefixed by the common suffix TAC, we know that the LCP value between these two rotations is at least 3. From Figure 4, we find that their remainders correspond to the third (index 2) and fourth (index 3) rotations of the ${BWT}_{P}$ matrix, so we need to perform an RMQ on the SLCP between the range of [2 + 1 · · · 3] and this returns 8, the only value within the range. Therefore, ${LCP}_{T}[23]=3-2+8=9$, which matches the value shown in Figure 2C. Note that *w* must be subtracted from the suffix length in this case, because the minimum SLCP value already accounts for the *w*-length overlap between the common suffix prefixing the rotations and the start of their remainders.

## PFP applications

PFP has been applied in a variety of contexts to overcome the limitations of traditional indexing on large highly repetitive data. Table 1 presents a selected subset of works that use PFP, broadly categorized by their contribution.

### Building fundamental bioinformatic data structures

As previously discussed, the ${BWT}_{T}$ (Boucher et al. 2019), ${SA}_{T}$ (Kuhnle et al. 2020), and ${LCP}_{T}$ (Rossi et al. 2022) have been built from the PFP output. Boucher et al. (2019) showed that PFP enables substantially more resource-efficient construction of the run-length FM-index (RLFM-index) than Bowtie’s (Langmead et al. 2009) FM-index-based approach on large collections of human Chromosome 19 (Chr 19) sequences. Across collections of up to 1000 Chr 19 sequences, the RLFM-index was consistently at least an order of magnitude faster to build than Bowtie’s index and, once the collection exceeded 50 sequences, required an order of magnitude less peak memory. In both cases, the performance gap widened as additional sequences were added. On a 512 GB server, Bowtie exhausted all available memory after indexing just 250 sequences, whereas the PFP-based RLFM-index was successfully constructed for all 1000 sequences within the same memory constraints. Additional evidence supporting these claims is presented in the Experimental results section. Subsequently, both Kuhnle et al. (2020) and Rossi et al. (2022) further demonstrated the efficiency of PFP-based construction algorithms for large, repetitive data sets.

To further reduce both the time and memory requirements of constructing the aforementioned data structures for large data sets, recursive PFP has been applied (Oliva et al. 2023; Ferro et al. 2024) (see the Recursive prefix-free parsing section for details). Oliva et al. (2023) showed that, for extremely large collections of sequences, constructing the ${BWT}_{T}$ from the output of recursive PFP is more computationally efficient than the construction from standard PFP. They repeated the experiment of Boucher et al. (2019), this time scaling up to 2400 Chr 19 sequences, more than double the amount used previously. Compared with the standard PFP-based construction, the recursive PFP-based approach reduced memory usage from approximately 40 to 15 GB and halved the running time from roughly 3000 to 1500 s, demonstrating substantial improvements in both speed and memory efficiency. However, this improvement only started to become noticeable after around 500–1000 sequences. For smaller collections, the performance difference between the two methods was negligible. These empirical results are supported by the theory of recursive PFP. Ferro et al. (2024) extended this work and showed how to build the ${SA}_{T}$ and ${LCP}_{T}$ alongside the ${BWT}_{T}$ with recursive PFP.

### Extensions and specialized indexing structures

Beyond the classical bioinformatics data structures discussed in this paper, PFP has also been used to build extensions of these structures, or components thereof, including the compressed suffix tree (CST) (Boucher et al. 2021b; Oliva et al. 2022), the extended BWT (eBWT) (Boucher et al. 2021a, 2024), the suffixient set (Cenzato et al. 2024), and the move structure (Zakeri et al. 2024, 2025). Rather than directly building the CST, Boucher et al. (2021b) show that the output of PFP can be augmented to support full suffix tree functionality. This is particularly important because the suffix tree can be viewed as a unifying conceptual framework for many string data structures, including those considered in this paper, and therefore plays a central role in bioinformatics, at least from a historical point of view. Suffix trees have a large space overhead relative to the input, and although CSTs reduce this overhead, they remain expensive to construct. Notably, the PFP-based CST construction by Boucher et al. (2021b) was shown to be more time and memory efficient than the state-of-the-art implementations in the Succinct Data Structure Library (SDSL) (Gog et al. 2014), a widely used benchmark library for compressed data structures. The eBWT extends the BWT to string collections by grouping related subsequences across different strings (Mantaci et al. 2007). The eBWT enables similarity detection and has been used as an index in metagenomic applications, helping to determine the taxonomic origin of sequencing reads (Ander et al. 2013; Guerrini et al. 2020). The suffixient set (Depuydt et al. 2023; Cenzato et al. 2024; Navarro et al. 2025) is a novel data structure that stores a small subset of integers capturing all branching points of a suffix tree. Although being new, it holds promise as a compressed index for string matching tasks in bioinformatics. The move structure (Nishimoto et al. 2022) is another new compressed data structure, based on the BWT. It has the unique beneficial properties of being stored in O(r) space while still supporting O(1) LF queries. Zakeri et al. (2024, 2025) implemented the data structure and showed that, in practice, its favorable locality of reference limits cache misses, leading to significant speed gains for string matching queries over other pangenomic data structures, such as the *r*-index.

### String matching queries

Another subset of PFP research focuses on string queries enabled by data structures constructed from PFP. Many useful string matching queries can be calculated from matching statistics. Briefly, the matching statistics *MS*[0 · · · *m* − 1] describe how a read *R*[0 · · · *m* − 1] aligns to a text *T*[0 · · · *n* − 1]. For each position *i* in *R*, *MS*[*i*] returns a pair: the length of the longest prefix starting at *R*[*i*] that occurs in *T*, and the position of one such occurrence in *T*. Matching statistics have been used in PFP applications to support various string queries, including maximal exact matches (MEMs) (Rossi et al. 2022), which cannot be extended in either direction; maximal unique matches (MUMs) (Giuliani et al. 2022; Shivakumar and Langmead 2025), which occur only once in *T*; and pseudo matching lengths (PMLs) (Ahmed et al. 2021, 2023), an efficient approximation of matching statistics. Varki et al. (2025) used MEMs as seeds for their *r*-index based short-read aligner, adopting the approach popularized by BWA-MEM (Li 2013). Shivakumar and Langmead (2025) computed multi-MUMs across 300+ human assemblies using under a terabyte of memory, facilitating the visualization of novel structural insights. Ahmed et al. (2021, 2023) demonstrated that PMLs can be computed in a streaming manner fast enough to process Nanopore sequencing reads in real time, allowing for direct metagenomic classification.

### Text compression and grammar induction

A further line of PFP research investigates its use as a preprocessing step to construct classical compressed representations, building on its origins in text compression. Hong et al. (2023) showed that PFP can be used to construct the LZ77 factorization, a compression algorithm that divides the text into the longest substrings matching earlier occurrences (Ziv and Lempel 1977). LZ77 is a foundational algorithm in string processing and forms the basis of many modern compression algorithms, including LZMA2 and Deflate (core algorithm of gzip). Similarly, Gagie et al. (2019) showed that PFP (called CTPH at the time) can be used to build a variant of the RePair grammar. RePair is a grammar-based compression algorithm that recursively replaces the most frequent adjacent pair of characters with a new previously unseen character until no repeats remain (Larsson and Moffat 2000). Although conceptually simple, it achieves good compression in practice. However, it requires memory proportional to the input size, making it impractical for large data sets. Gagie et al. (2019) showed that their PFP-based RePair grammars can be constructed for inputs several orders of magnitude larger than those handled by standard RePair, using a fraction of the time and memory. Kim et al. (2024) extended this work and showed that both time and memory usage could be further reduced when the grammar was built from recursive PFP, noting that these benefits were observed only for large collections of sequences, consistent with the findings of Oliva et al. (2023).

## Experimental results

**Figure 5.**
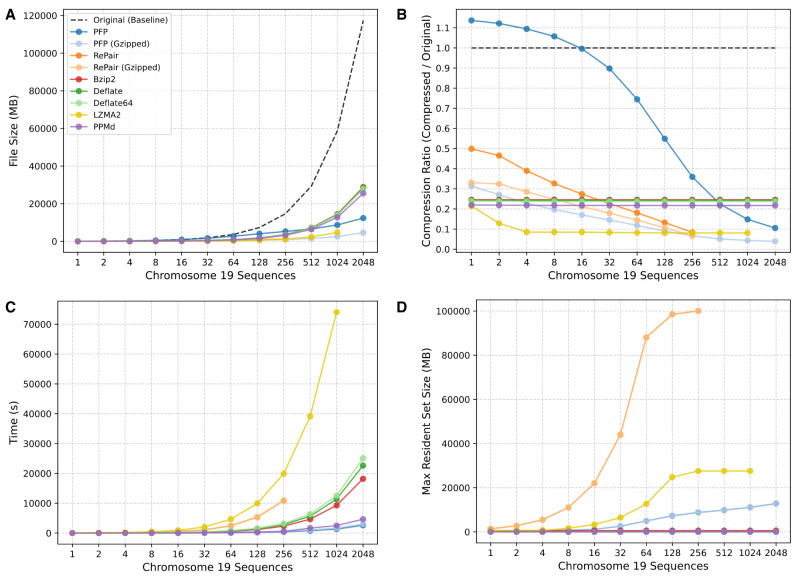
Performance comparison of PFP against standard compressors on an increasing number of human Chromosome 19 sequences. The subplots show: (*A*) compressed file size (MB), (*B*) compression ratio (defined as compressed/original size), (*C*) wall-clock time (s), and (*D*) peak memory usage (MB). All files were compressed using a single thread.

We benchmarked PFP^1^ against a grammar-based compressor (RePair^2^) and general-purpose compressors^3^ (Bzip2, Deflate, Deflate64, LZMA2, and PPMd) on an increasing number of human Chromosome 19 (Chr 19) sequences from the 1000 Genomes Project (The 1000 Genomes Project Consortium 2015). These files were used without any preprocessing, retaining their headers and whitespace. As discussed in the Prefix-free parsing section, PFP was designed as a preprocessing algorithm for building other data structures in a memory-efficient manner. As a result, its output (the dictionary and parse) does not natively exploit the benefits of advanced compression techniques such as entropy coding or bit-packing, unlike the general-purpose compressors whose primary goal is compression. To evaluate the compressibility of the PFP output, we archived and compressed its dictionary and parse using tar and gzip (denoted as PFP (GZipped)). We applied the same procedure to the RePair output. PFP was run with its default parameter settings (*w* = 10, *p* = 100). All the general-purpose compressors were run at compression level 6 (on a scale of 1–9, where 1 denotes minimal compression and 9 maximal), which is the default or near-default setting. All files were compressed using one thread. All experiments were run and benchmarked with Snakemake (v7.32.4) (Mölder et al. 2025) on a server with an 2.95 GHz AMD EPYC 75F3 32-core processor. All tasks were allocated up to 100 GB of memory and a 24-h runtime, and were terminated if either limit was exceeded. The benchmarking results can be seen in Figure 5.

The experimental results confirm that PFP is a highly efficient compressor when run on large repetitive data sets. As shown in Figure 5A, PFP compressed all 2048 Chr 19 sequences (117.4 GB) to the smallest size (12.3 GB) among the tested compressors. This is reflected in Figure 5B as PFP has the smallest compression ratio among all the compressors at 0.1 for the full set of sequences. For context, compression ratio is defined as compressed size divided by uncompressed size. These numbers further improved when the PFP output was gzipped, reducing the file size to 4.6 GB and the compression ratio to 0.04. For comparison, Deflate achieved a compression ratio of 0.24 on the full set. Because Deflate is the underlying algorithm used by gzip, the fact that PFP (Gzipped) achieved over an 80% reduction in file size suggests that the PFP output is significantly more compressible than the original text. The only compressors that appeared to rival PFP on the full set were RePair and LZMA2; however, neither was able to compress the entire data set under the given constraints as LZMA2 timed out and RePair ran out of memory (see Fig. 5C,D). The motivation for using PFP on specifically large data sets is evident from Figure 5B. For the first 16 sequences, PFP had a compression ratio above 1.0, meaning its output was larger than the input. This occurs when there is little phrase repetition among the sequences, causing both the dictionary and parse to grow proportionally with the number of input sequences. However, with enough sequences, the dictionary size was observed to grow sublinearly, causing the compression ratio to decrease.

The computational performance benchmarks show that PFP performs favorably compared to the other compressors, with increasing advantages as the data set size grows. As shown in Figure 5C, PFP was the fastest method, compressing all 2048 Chr 19 sequences in 2612 s (1.28 s/sequence). The only method with comparable speed was PPMd, which compressed the full set in 4638 s (2.26 s/sequence), nearly twice as long as PFP. PFP is fast because it processes the input in a single pass using a sliding window with rolling hashes, avoiding expensive backtracking or repeated scans of the data. In terms of memory usage, PFP was efficient, though not the most memory efficient among the compressors. As shown in Figure 5D, PFP used 12.8 GB to compress all the Chr 19 sequences, representing only a small fraction (0.11) of the uncompressed data set size. Moreover, PFP required significantly less memory than LZMA2 and RePair, which used 27.6 and 100 GB, respectively, on their last completed data set. By contrast, Deflate, Deflate64, BZip2, and PPMd used very little memory (<1 GB). The reason the memory usage increases in PFP is because the dictionary and parse are kept in memory throughout the parsing.

The compression and computational benchmarks together demonstrate that PFP is a practical and competitive choice for compressing large, repetitive data sets like biological data sets.

## Future directions

As described in the PFP applications section, PFP has been used in a wide range of applications. Despite its adoption, there are still opportunities to refine PFP and develop new applications that leverage its capabilities. One potential improvement to PFP lies in the way the dictionary is represented. PFP achieves its best compression on large collections of highly similar sequences, as repeated identical phrases cause the dictionary to grow sublinearly compared to the parse. However, when a variation is encountered, the entire phrase containing it must be added to the dictionary, even if it differs only by one character from another existing phrase (Oliva et al. 2022). Seemingly, new phrases caused by variations to existing phrases could be represented more space-efficiently in the dictionary.

A recent paper by Díaz-Domínguez et al. (2025) presents a method to merge BWTs constructed with PFP. This is significant because it potentially enables parallel BWT construction via multithreading. Their key insight is that if trigger strings in each partition are unique across partitions, then phrase suffixes remain unique to their respective partitions. As a result, the relative order of the characters in the BWT of each partition is preserved in the merged BWT, because no ties occur between suffixes of different partitions. The merged BWT is then constructed by lexicographically comparing the suffixes between partitions and writing the corresponding characters accordingly. The challenge is to find unique trigger strings that only appear in specific partitions that are valid throughout the text. To find this set of trigger strings, they parse each partition and remove any triggers that appear in other partitions. This approach seems impractical for collections of similar texts, where trigger strings are likely shared. The authors acknowledge this limitation and note that it performs better on diverse text collections. However, the compression benefits of PFP are reduced with more diverse texts, highlighting the need for a better solution.

In addition to building data structures, PFP may help improve the performance of large language models (LLMs), which are increasingly being studied and applied in bioinformatics (Ji et al. 2021; Nguyen et al. 2023; Zhang et al. 2023; Sanabria et al. 2024; Zhou et al. 2024). An important yet understudied aspect of these models is how their input tokens are defined. Tokens are small units of data that collectively form the input of the model. They are essential for reducing the input space, which would be prohibitively large if the model processed the data directly. The choice in tokenizer has been shown to directly influence model performance (Dotan et al. 2024; Lindsey et al. 2025). Currently, *k*-mer and byte-pair encoding (BPE) (Gage 1994; Sennrich et al. 2016) are the most widely used tokenization schemes in bioinformatics-specific LLMs, but both have limitations. *k*-mer tokenization divides the input sequence into overlapping subsequences, each consisting of *k* consecutive characters. A key limitation is that individual tokens often lack a meaningful representation of the underlying data. Moreover, *k* cannot be set too large due to the limited vocabulary capacity of these models. In contrast, BPE is a dynamic tokenization method that is a variation of the RePair algorithm (briefly discussed in the PFP applications section), but it merges the most frequent adjacent characters or existing tokens until a target vocabulary size is reached. However, BPE can introduce intermediate scaffold tokens that become part of the vocabulary but are rarely used. PFP has the potential to address both issues. By treating PFP-generated phrases as tokens, it produces a minimal set of variable-length tokens that occur at least once in the input, which can be fine-tuned by changing the *w* and *p* parameters.

## Conclusion

PFP has emerged as a valuable preprocessing technique for scalable construction of key bioinformatics data structures. As biological data sets continue to grow in size and complexity, PFP is poised to play an increasingly critical role due to its ability to scale efficiently with the size of the data. It has already demonstrated the capability to process hundreds of gigabytes of data. Beyond its practical utility, PFP provides a robust theoretical framework that enables researchers to extend its use, enabling a wide range of applications built on it. Although its primary adoption has been within bioinformatics, PFP is a generalizable algorithm that performs effectively on highly repetitive data sets. Its versatility and scalability position PFP as a foundational approach to the efficient construction of data structures on repetitive data sets.

## Competing interest statement

The authors declare no competing interests.

## Footnotes

1
https://github.com/marco-oliva/pfp

2
http://www.dcc.uchile.cl/gnavarro/software/repair.tgz

3 All provided by 7-Zip, a popular open-source archiver tool (https://www.7-zip.org).
